# Supplementary material for: Identification of Cancer Cells in the Human Body by Anti-Telomerase Peptide Antibody: Towards the Isolation of Circulating Tumor Cells
Source: Int J Mol Sci. 2022 Oct 25;23(21):12872. doi: 10.3390/ijms232112872 (PMC9654147; doi:10.3390/ijms232112872)
Supplement: Supplementary file 1 [file ijms-23-12872-s001.zip › ijms-1970921-supplementary.pdf]

Supplementary figure legends

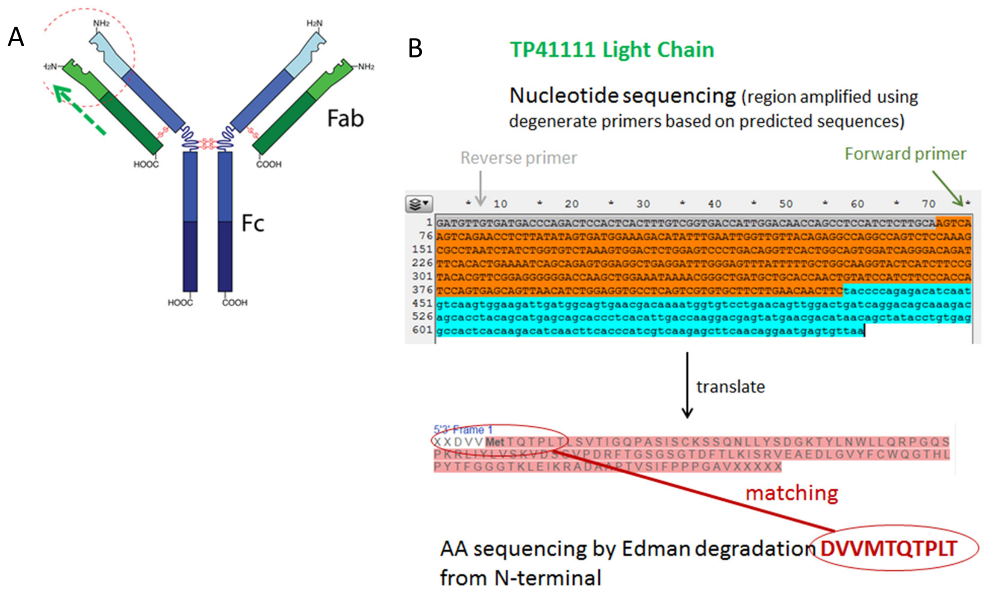

Supplementary Figure S1. The αhTERT mAb binds ovarian carcinoma

(A). The αhTERT mAb binds to ~ 4% of cells isolated from surrounding ovarian carcinoma tissues. The binding of the α–hTERT mAb was analyzed by flow cytometry as described in the previous figures.

(B). The α–hTERT mAb binds to ovarian tumor cells isolated from a patient with ovarian carcinoma, partial co-expression with CCR5+. The α-hTERT mAb or its isotype control were added to ovarian tumor cell suspension. hTERT positive cells were visualized by FITC labeled α-mouse secondary Ab, 4% of ovarian tumor site derived cells were α-hTERT positives. After washing AF647-CCR5 or control IgG2b k isotype was added. The binding was analyzed by flow cytometry and is presented as FlowJo overlay dot plot of samples with the same number of cells colored according to samples. Double positive αhTERT+/CCR5+ cells are shown in red, control isotype IgG1/ IgG2b control cells are in green. 60-97% of α-hTERT+ cells are CCR5+.

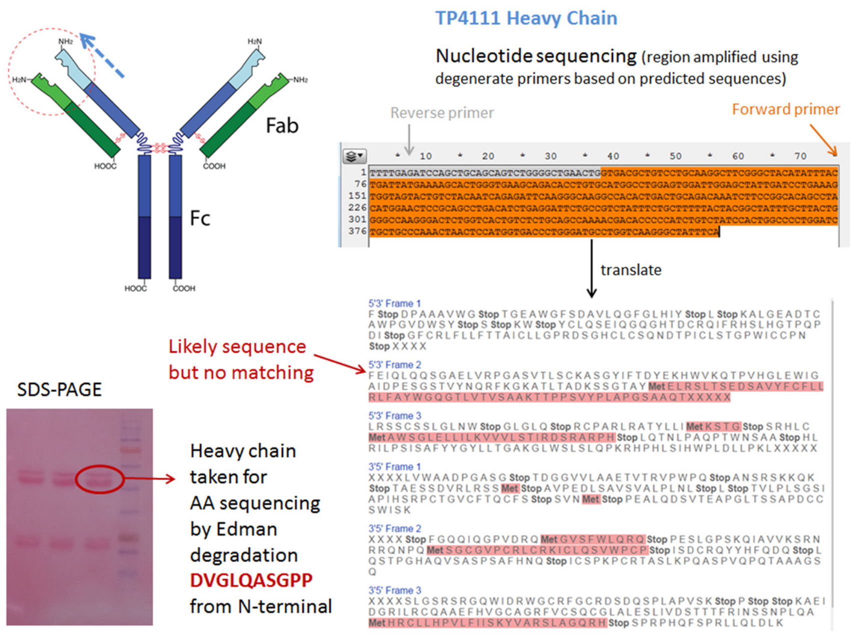

Supplemented Figure S2. The α–hTERT mAbs Binds circulating neoplastic cells obtained from a patient with Merkel cell carcinoma (a case study). PBMCs of Merkel carcinoma patient were incubated with the α-hTERT mAb or its isotype IgG1, washed and stained with FITC α mouse secondary Ab, then analyzed by flow cytometry. Histogram overlay: the isotype is marked in green, αhTERT mAbs - in red and violet.

A

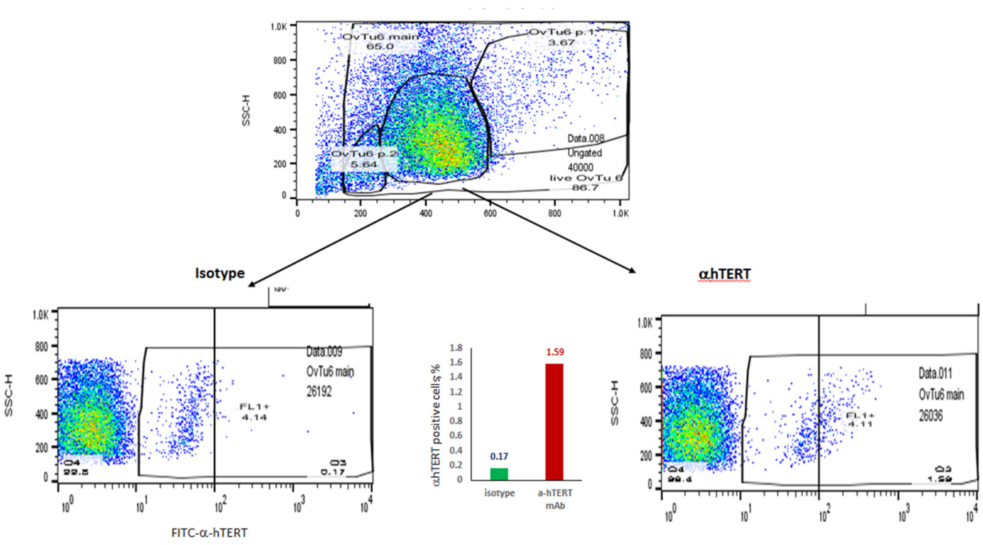

B

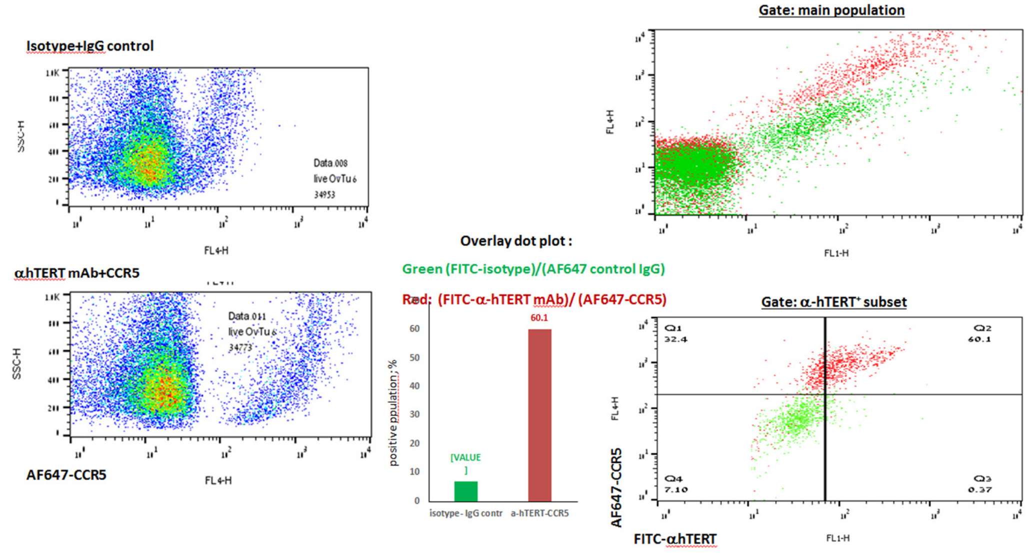

Supplementary Figure S3. The α-hTERT mAb Binds breast cancer cells.

The α-hTERT mAb binds cells obtained from a patient with a breast tumor (a case study). Breast tumor suspension cell aliquots were incubated with α-hTERT mAb or its isotype IgG followed by staining with FITC-α-mouse secondary Ab. Live cells are gated. The dot plots present a clear shift of the subpopulation in samples stained by the α-hTERT mAb.

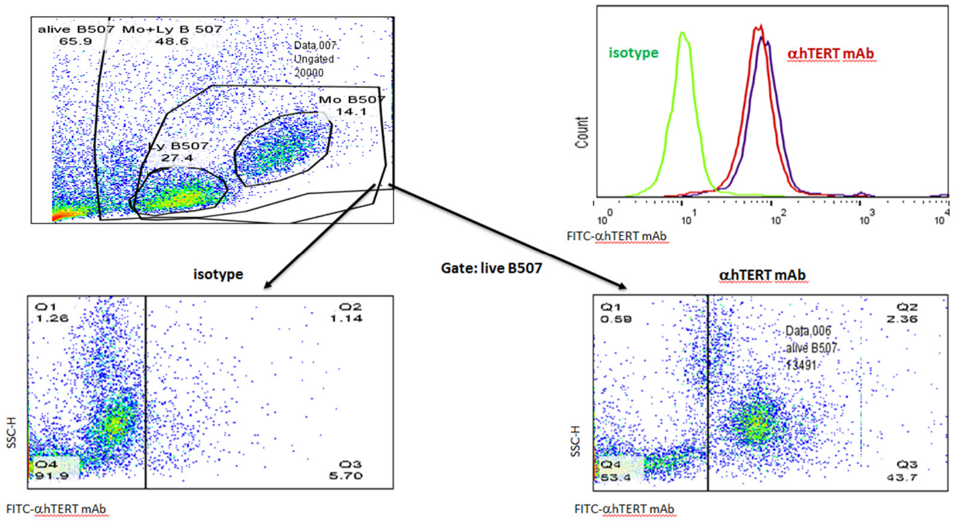

Supplemented Figure S4. The α-hTERT mAb binds to PBMCs from patient with lung cancer (a case study). The binding of the α-hTERT mAb to blood circulating lung tumor cells was analyzed by flow cytometry. Overlay histogram: the isotype is marked with green, the α-hTERT mAb – with red.

A

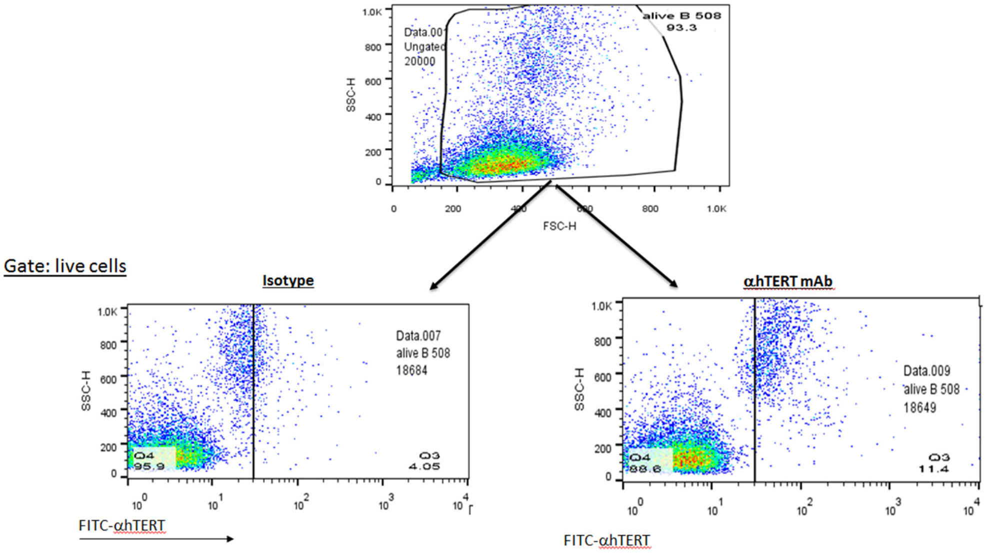

B

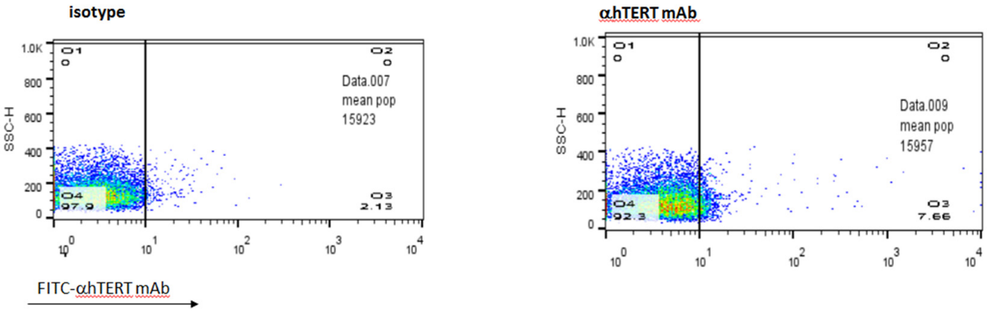

Supplemented Figure S5. The light chain of the αhTERT mAB.

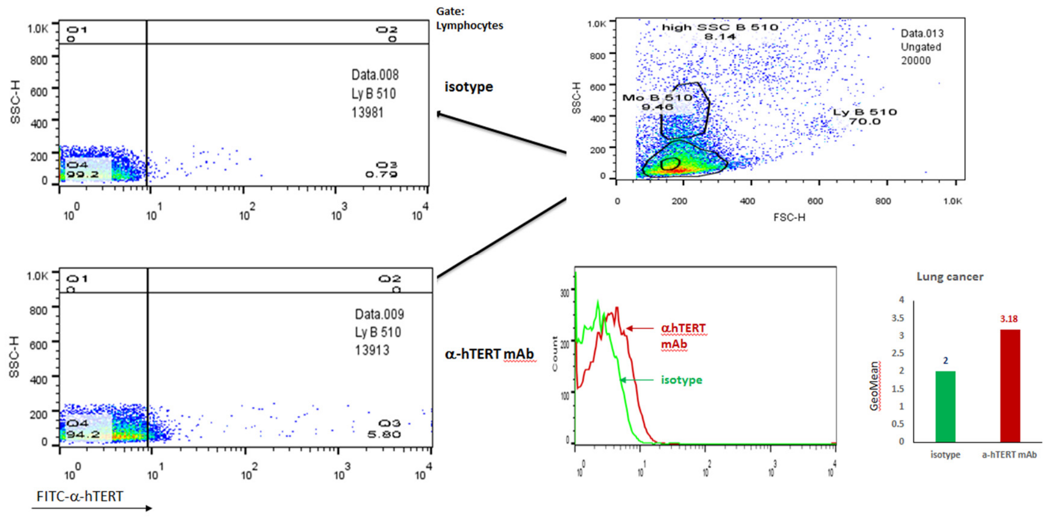

Supplemented Figure S6. The heavy chain of the αhTERT mAB.
